# Strain driven phase transition and mechanism for Fe/Ir(111) films

**DOI:** 10.1038/s41598-021-01474-1

**Published:** 2021-11-09

**Authors:** Chen-Yuan Hsieh, Pei-Cheng Jiang, Wei-Hsiang Chen, Jyh-Shen Tsay

**Affiliations:** 1 Department of Physics, National Taiwan Normal University, Taipei, 116 Taiwan; 2 Department of Electronic Engineering, Minghsin University of Science and Technology, Hsinchu, 30401 Taiwan

**Keywords:** Magnetic properties and materials, Phase transitions and critical phenomena, Surfaces, interfaces and thin films, Condensed-matter physics

## Abstract

By way of introducing heterogeneous interfaces, the stabilization of crystallographic phases is critical to a viable strategy for developing materials with novel characteristics, such as occurrence of new structure phase, anomalous enhancement in magnetic moment, enhancement of efficiency as nanoportals. Because of the different lattice structures at the interface, heterogeneous interfaces serve as a platform for controlling pseudomorphic growth, nanostructure evolution and formation of strained clusters. However, our knowledge related to the strain accumulation phenomenon in ultrathin Fe layers on face-centered cubic (fcc) substrates remains limited. For Fe deposited on Ir(111), here we found the existence of strain accumulation at the interface and demonstrate a strain driven phase transition in which fcc-Fe is transformed to a bcc phase. By substituting the bulk modulus and the shear modulus and the experimental results of lattice parameters in cubic geometry, we obtain the strain energy density for different Fe thicknesses. A limited distortion mechanism is proposed for correlating the increasing interfacial strain energy, the surface energy, and a critical thickness. The calculation shows that the strained layers undergo a phase transition to the bulk structure above the critical thickness. The results are well consistent with experimental measurements. The strain driven phase transition and mechanism presented herein provide a fundamental understanding of strain accumulation at the bcc/fcc interface.

## Introduction

The use of ultrathin layers of transition metals on substrates is a subject that has been of considerable interest in the past decades because of technological applications in enhancing catalytic activities, the formation of surface alloys, spintronic materials, biosensor application, and two-dimensional materials^1–21^. For example, layered planar and buckled phases of silicene are prepared in a self-organization process during annealing of ultrathin layers of Au grown on Si(111) surface^1^. The exposure of Ag/PdAg/Ag(111) islands to CO at room temperature induces the migration of Pd to the surface, a process that is driven by the energetic stabilization of the Pd–CO bond^4^. The larger (smaller) atomic size of a Pb (Ge) atom with respect to an Ag atom results in the formation of commensurate (incommensurate) interfaces and further demonstrates that the splitting bands of the Ag_2_Ge surface alloy and a one-monolayer (ML) Pb film originated from the common incommensurate interface with Ag(111)^6^. In terms of modifying the contact area of the metallic/magnetic interface, a method for preparing magnetic layers with different levels of enhanced magnetic anisotropy energy was developed for Ag ultrathin overlayers on an annealed Ni/Ag interface^7^. A phase transition of a superparamagnetic phase can be induced by controlling the thickness of the ultrathin ferromagnetic layers of Co/Ir(111) and the larger total magnetic moments as compared to nanoparticles that pave the way for the further development of strategies for fabricating biosensors on a microchip^8^. After an annealing treatment for a 5 ML Fe/Pt(111) layer to form a surface alloy, the coercive force is greatly enhanced by as much as 1100%, and the resulting magnetic properties are strongly related to the diffusion of Fe atoms into the Pt(111) substrate^14^. A spin reorientation transition was observed for Fe/Pt(111) ultrathin films by introducing a Ag overlayer as thin as 1 ML^15^. In terms of introducing heterogeneous interfaces, the stabilization of crystallographic phases is critical to a viable strategy for the development of new materials with novel characteristics^17–25^. For example, Fe_50_Mn_50_ epitaxial films grown on GaAs(001) were found to undergo a structural transition from a face-centered-cubic (fcc) phase to a body-centered-cubic (bcc) phase which does not exist in nature^18^. For bcc-Fe_50_Mn_50_ epitaxial films, the occurrence of ferromagnetism is accompanied by a structural phase transition due to the tensile strain at the interface^18^. An anomalous enhancement in the total magnetic moment and changes in coercivity were previously reported for Ni_x_Ti_1−x_/Ni and Ni_x_Ti_1−x_/Co, changes that can be attributed to interfacial strain, as evidenced by the magnetoelastic coupling in these heterostructures^19^. In the case of the diffusion of hydrogen through a Pd nanoparticle/Mg film interface, the Pd undergoes a strain-induced phase transformation near the interface, and the saturation of the Pd nanoparticles with hydrogen enhances their efficiency as nanoportals^21^.

Iron and iron-based alloys are the most important materials used in magnetic devices, such as magnetic tunnel junction^26–28^, magnetic random access memory^29,30^ and magnetic sensors^31–33^. Because of their different lattice structures, Fe/Ir interfaces function as a platform for controlling pseudomorphic growth, nanostructure evolution, and the formation of strained clusters. For iron monolayers that are adsorbed on a vicinal iridium surface, the maximum local magnetic moment is located at the edge atom, whereas the least is at the kink atoms^34^. For Ir*/*FeCo bilayers on MgO, the Ir cap layer can induce both a large perpendicular magnetic anisotropy (PMA) and a colossally high voltage controlled magnetic anisotropy efficiency by the strain introduced at the interface^35^. The Fe monolayer tends to be antiferromagnetic for iron on Ir(100) with a relaxed geometry, while an unrelaxed Fe/Ir(001) sample exhibits a ferromagnetic ground state^36^. As the Fe thickness increases for Fe/Ir(111), the pseudomorphic growth of Fe films was observed followed by the bcc(110) arrangement of Fe atoms into a Kurdjumov–Sachs orientation^10,11^. After the annealing of fcc-Fe/Ir(111) at high temperatures, a layered structure composed of some Fe atoms on the top of a Fe_0.5_Ir_0.5_ interfacial alloy is formed, which can be attributed to competition between the negative heat of formation of Fe_0.5_Ir_0.5_ and the surface free energy of Fe^9^. Recent research studies regarding the Dzyaloshinskii–Moriya interaction (DMI) for Fe/Ir(111) show the formation of magnetic textures consisting of chiral spin-spirals and skyrmions^37,38^. Although a large number of efforts have been devoted to the exploration of Fe/Ir interfaces, our knowledge regarding the strain accumulation phenomenon in ultrathin Fe layers on fcc substrates remains somewhat limited. We focus herein on the structures and the accumulation of strain for ultrathin Fe/Ir(111). The interfacial strain energy was calculated for different Fe thicknesses by converting the interlayer distance and in-plane lattice parameters into lattice parameters in cubic geometry. The results of the calculation, which are consistent with the experimental measurements, indicate that, above a critical thickness, the strained layers undergo a phase transition to a bulk structure.

## Experimental

The preparation and characterization of all samples were carried out in an ultrahigh vacuum (UHV) chamber with a base pressure below 2 × 10^−10^ mbar. The Ir(111) substrate was prepared by cycles of Ar^+^ ion bombardment and annealing treatments. Before each experiment, the presence of a well-ordered p(1 × 1) structure was confirmed using low-energy electron diffraction (LEED) techniques. The purity of the sample was checked by Auger electron spectroscopy (AES). Iron atoms were evaporated from a well-collimated evaporator where high purity (99.997%) Fe rods were contained in a crucible. The deposition rate was determined by means of the ratio of the intensity of the Fe and Ir Auger signals and was further checked by oscillations of the specular beam of LEED^10,39^. The surface of the iridium single crystal was cleaned after each run. A He–Ne laser with a wavelength of 632.8 nm was used as the light source for the magneto-optical Kerr effect (MOKE) measurements. The magnetic field was applied in-plane and perpendicular to the surface of the sample in the longitudinal and polar configurations, respectively. Experimental details and various components have been described in detail elsewhere^8–10,39^.

## Results and discussion

**Figure 1:**
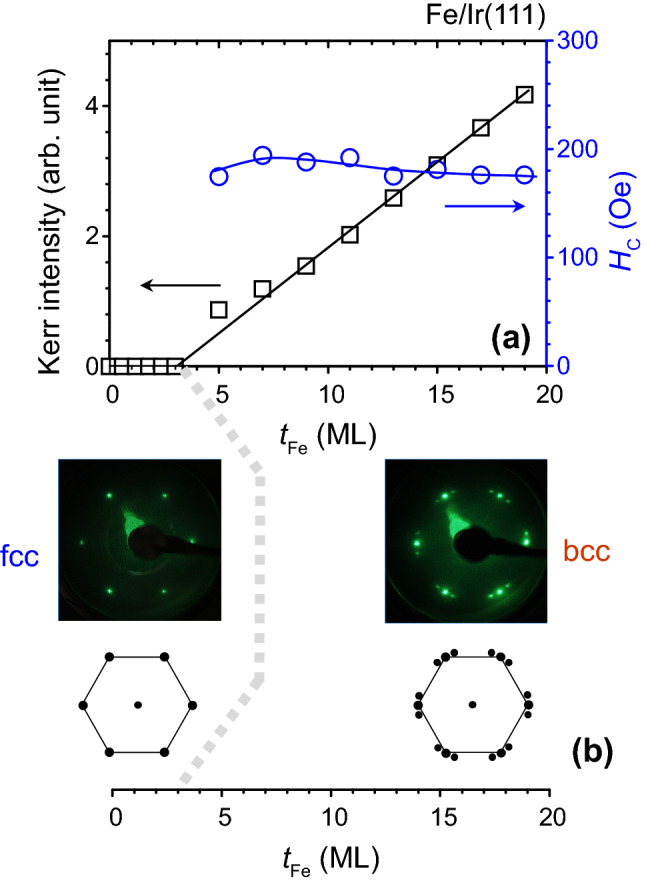
(**a**) Kerr intensity and coercivity versus Fe thickness in the longitudinal configuration. (**b**) LEED patterns for fcc-Fe and bcc-Fe at different thickness regimes.

Figure 1a shows both the Kerr intensity and coercive force versus the Fe thickness (*t*_Fe_) as Fe atoms are deposited on the Ir(111) surface. For a Fe layer thinner than 3 ML, the Kerr intensity exhibits a zero value, indicating nonmagnetic behavior of the Fe layer. By increasing the Fe thickness, the linear characteristics of the Kerr intensity for Fe layers thicker than 3 ML show that the Curie temperature is well above the measurement temperature of 300 K. The straight-line extrapolation of the data for Fe thicknesses well above 3 ML to the zero signal passes through a Fe thickness of roughly 3 ML. This result shows that the first 3 ML of Fe are a magnetic dead layer. From the literature, both imperfections and magnetocrystalline anisotropy give rise to hysteresis as well as a coercive force^40^. For 5 ML Fe/Ir(111), the coercivity is around 175 Oe. As the Fe thickness increase, the coercivity slightly increases to around 190 Oe (7 ~ 11 ML) and decreases to 175 Oe (> 13 ML). The reduction in coercive force could be attributed to both smaller magnetocrystalline anisotropy and fewer imperfections in the films. From LEED studies for Ir(111) substrate, a p(1 × 1) structure of bright spots in hexagonal arrangement is observed. For Fe/Ir(111) thinner than 3 ML, the same p(1 × 1) structure is observed (Fig. 1b) using LEED technique showing pseudomorphic growth of Fe films^9,10^. From Fig. 1a, b, the ferromagnetic dead layer coincides with the formation of fcc(111)-Fe for the first three MLs of Fe/Ir(111). For Fe/Ir(111) films thicker than 3 ML, additional spots surrounding each primary beam are observed (Fig. 1b). The additional spots can be identified to be related to the bcc(110) arrangement of Fe atoms in Kurdjumov–Sachs (KS) orientation of bcc(110)/fcc(111) systems^9,10^. The KS model is a special case for the one-dimensional matching between bcc(110) and fcc(111) lattices where the bcc[$\overline{1 }$11] direction is parallel to the fcc[10$\overline{1 }$] direction. The unit cell of Fe bcc(110) with quasi-hexagonal shape and shorter edges of 0.248 nm forming angles of 70.5° and 109.5°. Because of the threefold symmetry of the surface layer for Ir(111), one can obtain the LEED pattern for KS orientation by rotating 120° and 240° the reciprocal lattice of bcc(110). Detailed discussions for the KS orientation of Fe/Ir(111) can be found in the reference 10. The linear increase in the Kerr intensity versus the Fe thickness is attributed to the growth of a bcc(110)-Fe phase.

**Figure 2:**
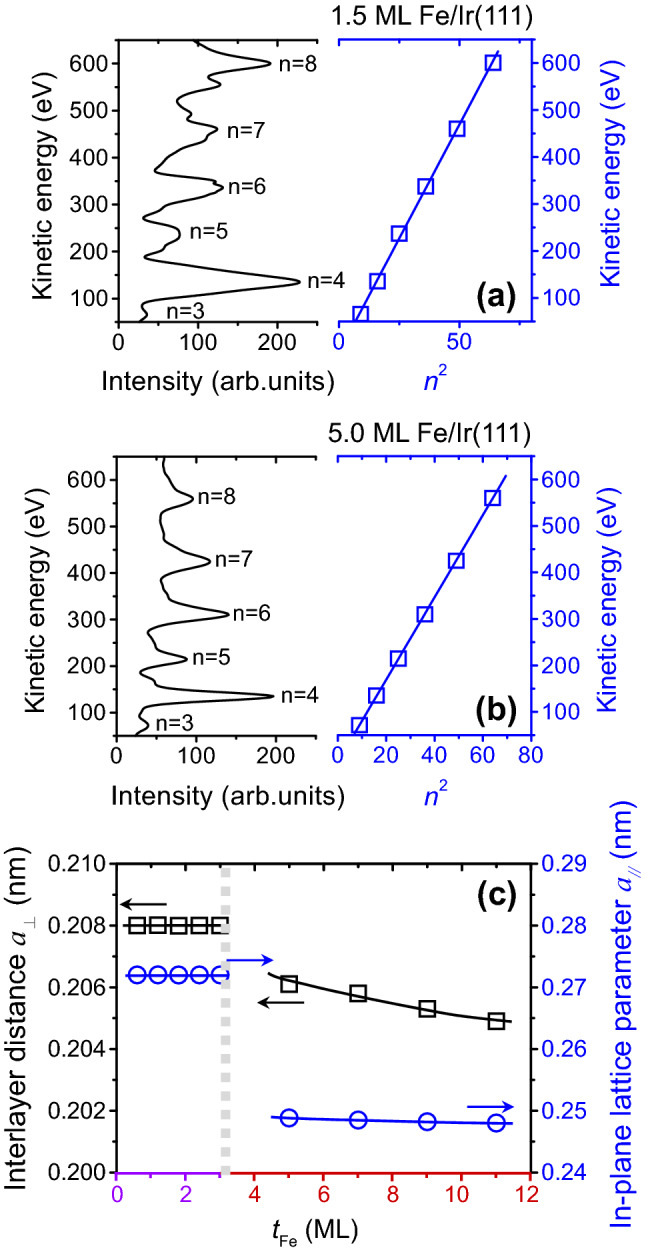
LEED *I–V* curves and *E–n*^2^ plots of (**a**) 1.5 ML Fe/Ir(111) and (**b**) 5.0 ML Fe/Ir(111). (**c**) Interlayer distance $a_{ \bot }$ and in-plane lattice parameter $a_{//}$ versus Fe thickness.

From an analysis of LEED patterns, a structural phase transition occurs when the Fe thickness is increased for Fe/Ir(111). Considering the different structures of bulk iron and iridium, it would be predicted that strain is induced at the Fe/Ir(111) interface. In order to further analyze the strain accumulation in the films, detailed structural information on Fe/Ir systems was collected using a LEED technique. The interlayer distance $a_{ \bot }$ of the subsurface region can be derived from the *I*–*V* curve of the LEED specular beam. From the Bragg diffraction conditions

1 $$ 2a_{ \bot } \cos \theta = n\lambda = nh/\sqrt {2m_{e} E} , $$

the kinetic energy of the incident electrons *E* can be expressed by

2 $$ E = n^{2} \left( {\frac{{h^{2} }}{{8m_{e} a_{ \bot }^{2} \cos^{2} \theta }}} \right); $$

where *θ* is the angle between the incident electron beam and surface normal; *m*_e_ is the electron mass; *h* is Planck’s constant^10,16^. Figure 2a show a typical LEED *I–V* curve and *E–n*^2^ plot for Fe/Ir(111) with an fcc-Fe structure. The interlayer distance, ${a}_{\perp }$, can be calculated from the slope of the *E–n*^2^ plot. For 1.5 ML Fe/Ir(111), the interlayer distance ${a}_{\perp }$ was determined to be 0.208 nm. Systematical investigations show that the interlayer distance ${a}_{\perp }$ for Fe/Ir(111) with an fcc-Fe structure remains nearly constant for Fe/Ir(111) layers up to a thickness of 3 ML. Figure 2b show a typical LEED *I*–*V* curve and *E–n*^2^ plot for Fe/Ir(111) with a bcc-Fe structure. For 5 ML Fe/Ir(111), the interlayer distance ${a}_{\perp }$ was determined to be 0.249 nm by calculating the slope of the *E–n*^2^ plot. A smaller interlayer distance ${a}_{\perp }$ was determined for Fe/Ir(111) with a bcc-Fe structure as compared to that for an fcc-Fe structure. The interlayer distance ${a}_{\perp }$ versus Fe thickness is depicted in Fig. 2c. For Fe/Ir(111) with an fcc-Fe structure (*t*_Fe_ < 3 ML), the interlayer distance remains constant at around 0.208 nm. For thicker films with a bcc-Fe structure, the interlayer distance ${a}_{\perp }$ decreases slightly from 0.206 to 0.205 nm. From the literature, the in-plane lattice parameter for Ir(111) is ${a}_{\mathrm{Ir}}$=0.272 nm and this value can be used in the further analyses^9,10^. From the corresponding LEED patterns, the in-plane lattice parameters ${a}_{//}$ of fcc(111)-Fe and bcc(110)-Fe for different thickness regimes are also summarized in Fig. 2c. For Fe/Ir(111) with an fcc-Fe structure (*t*_Fe_ < 3 ML), the in-plane lattice parameters ${a}_{//}$ are 0.272 nm, very close to that of ${a}_{\mathrm{Ir}}$. By increasing the Fe thickness from 5 to 11 ML, the in-plane lattice parameter decreases slightly from 0.250 to 0.248 nm, a value close to the literature value for bulk Fe.

**Figure 3:**
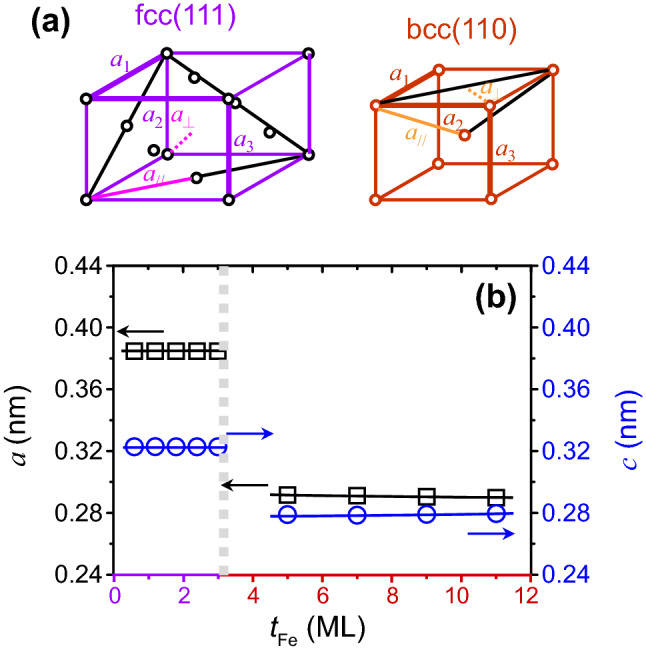
(**a**) Schematic diagrams of fcc(111) and bcc(110). (**b**) The lattice parameters *a* and *c* for fcc(111) and bcc(110) versus Fe thickness, by converting the experimental data for both interlayer distance ${a}_{\perp }$ and in-plane lattice parameters *a*_//_ to the lattice parameters *a* and *c*.

To further investigate the strain accumulation in the films, we converted the experimental data for both the interlayer distance ${a}_{\perp }$ and in-plane lattice parameters ${a}_{//}$ into the lattice parameters (*a*_1_, *a*_2_, *a*_3_) in cubic geometry for purposes of comparison. For Fe/Ir(111) layers thinner than 3 ML, Fe exhibits an fcc structure. The structural parameters of $a_{ \bot }$, $a_{//}$, and *a*_1_, *a*_2_, *a*_3_ are depicted in the left panel in Fig. 3a. By assuming *a*_1_ = *a*_2_ = *a* and *a*_3_ = *c*^23,24^, the relations between $a_{ \bot }$, $a_{//}$, and *a*, *c* can be expressed by

3 $$ a_{ \bot } = \frac{{\left| {a \cdot c} \right|}}{{\sqrt {a^{2} + c^{2} + c^{2} } }}, $$

4 $$ 2a_{//} = \sqrt {a^{2} + a^{2} } . $$

After substituting the experimental data for $a_{ \bot }$ and $a_{//}$ into Eqs. (3) and (4), the lattice parameters *a* and *c* versus the Fe thickness for distorted fcc-Fe can be calculated (Fig. 3b). For Fe overlayer thinner than 3 ML, the value for the lattice parameter *a* is nearly a constant at 0.384 nm, which is close to the bulk value for iridium in this thickness regime^41^. Fe/Ir(111) shows the pseudomorphic growth. A smaller value of 0.322 nm is obtained for the lattice parameter *c*. This shows that the strain induced at the Fe/Ir interface by the lattice mismatch strongly influences the structure of Fe and that this strain is different from that for the bulk structure. For Fe/Ir(111) thicker than 3 ML, the structure of Fe is restored to bcc. We assume *a*_1_ = *a*_2_ = *a* and *a*_3_ = *c*, and the relation between $a_{ \bot }$, $a_{//}$, and *a*, *c* can then be expressed as

5 $$ 2a_{ \bot } = \sqrt {a^{2} + a^{2} } , $$

6 $$ 2a_{//} = \sqrt {a^{2} + a^{2} + c^{2} } . $$

The lattice parameters *a* and *c* versus Fe thickness can be obtained (Fig. 3b). By increasing the Fe thickness for *t*_Fe_ > 3 ML, the lattice parameter *a* decreases slightly, approaching the bulk values of 0.287 nm for Fe^41^. This shows that the Fe lattice relaxes with increasing Fe thickness and approaches that for the bulk structure.

From an analysis of LEED results for Fe/Ir(111), a structural phase transition from fcc to bcc phase occurs when the Fe thickness is increased. Because of the different structures of bulk iron and iridium, the strain is induced at the Fe/Ir(111) interface. The strain energy can be obtained from the analysis of strain energy density where the bulk modulus and shear modulus as well as the experimental results of lattice parameters have to be put into calculations. In the following paragraphs, at first we discuss the strain driven phase transition (SDPT) model in terms of strain. At second, the interfacial strain energy is comparatively discussed with the surface energy in the limit distortion mechanism. Then we calculate the strain energy density from the analysis of elasticity in cubic structures and finally the relation between interfacial strain energy and Fe thickness is obtained.

**Figure 4:**
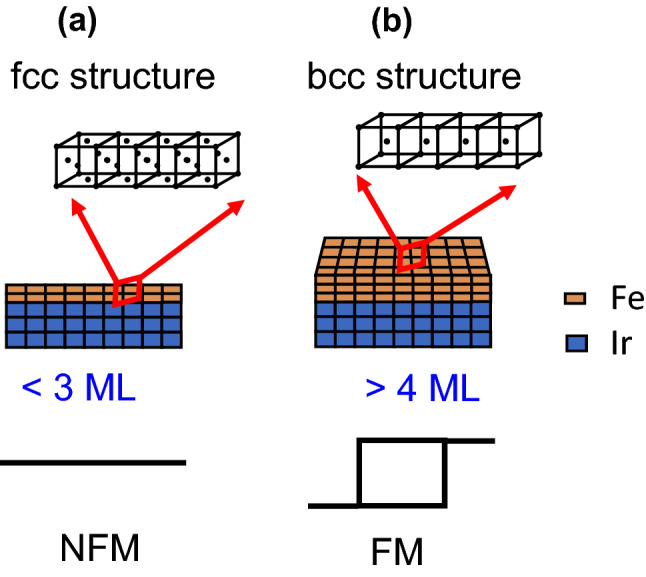
A schematic plot showing the SDPT model for describing the structural evolution and related magnetic phase transition for Fe/Ir(111) with thickness (**a**) < 3 ML, (**b**) > 4 ML.

Based on the magnetic measurements shown in Fig. 1 and the related structural evolutions in Figs. 1b, 2 and 3, we propose a SDPT model based on the experimental evidence showing that an fcc phase is transformed into a bcc phase. The SDPT model is schematically illustrated in Fig. 4. For a Fe/Ir(111) thickness thinner than 3 ML (Fig. 4a), Fe atoms on the Ir(111) substrate adopt an fcc structure. When the Fe thickness is increased, the interfacial strain energy increases while the films are nonferromagnetic. The accumulated strain may reach a certain value and the strained films then become unstable. For thicker Fe films (Fig. 4b), the overstrain condition in the films causes a structural change to a bcc phase while the phase transition leads to less stress/energetic stability. As a result of the structural phase transformation to bcc Fe films, the magnetic behavior changes to ferromagnetic for Fe/Ir(111) and this is the main concept of the SDPT model.

In order to explain the underlying driving force for the structural transition from an fcc phase to a bcc phase in the SDPT model, a limited distortion mechanism that takes the interfacial strain energy *E*_i_ and the surface energy *E*_s_ into account is proposed. As atoms are deposited on a substrate surface to form a heterogeneous interface, the atomic interactions mainly involve cohesive forces between adatoms, and the interfacial adhesive force between adatoms and the substrate. The cohesive force between adatoms is crucial for attaining a microscopic structure in a thermal equilibrium condition. From the energetic point of view, the cohesive force is related to the surface energy^42^. For a heterogeneous interface with a lattice mismatch, the storage of strain energy due to the lattice distortion occurs as the result of the competition between the adhesive force of adatoms and the interfacial adhesive force^43,44^. By increasing the thickness of the adlayer, the interfacial strain energy *E*_i_ increases accordingly and the storage of *E*_i_ may reach the value of the surface energy *E*_s_ at a critical thickness. In order to release the effects of the lattice distortion, the strained layers undergo a phase transition to the bulk structure above a critical thickness for Fe/Ir(111).

**Figure 5:**
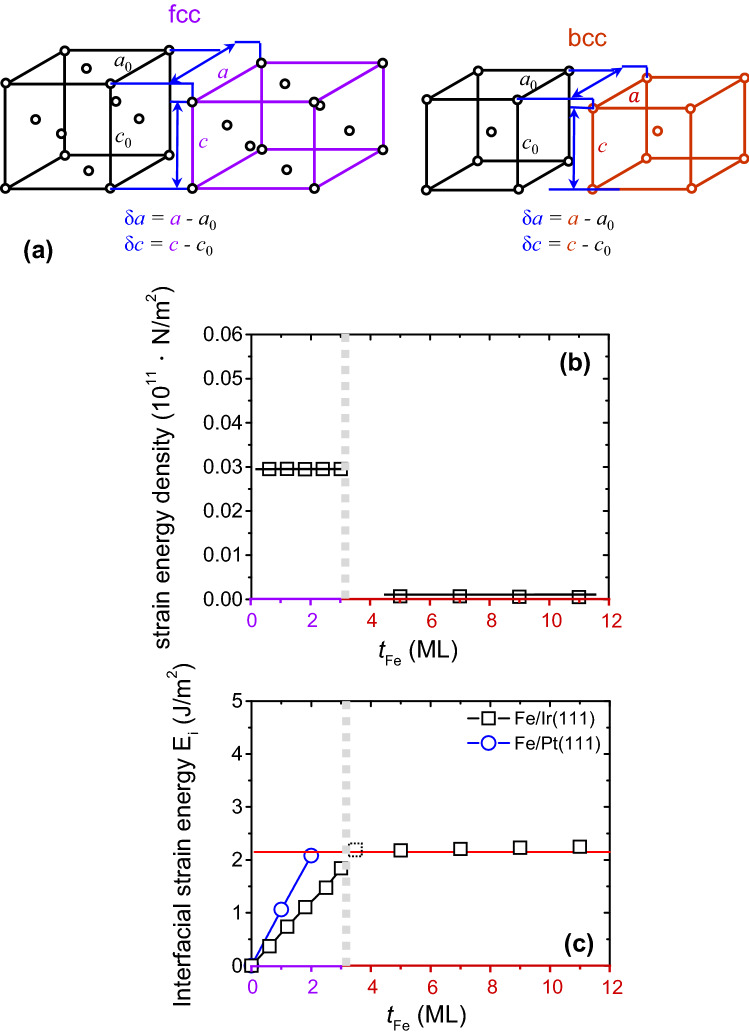
(**a**) Schematics of the original Fe bulk unit cells (black) and the distorted fcc (purple) and bcc (red) unit cells, where *a*_0_, *c*_0_ and *a*, *c* are defined as the lattice parameters for the original and distorted fcc and bcc unit cells, respectively. (**b**) Strain energy density and (**c**) interfacial strain energy versus Fe thickness for Fe/Ir(111) (black squares) and Fe/Pt(111) (blue circles).

Schematics of the original Fe bulk unit cells (black) and the distorted fcc (purple) and bcc (red) unit cells are shown in Fig. 5a; where *a*_0_, *c*_0_ and *a*, *c* are defined as the lattice parameters for the original and distorted fcc and bcc unit cells, respectively. From the analyses of the strain energy density around a cubic structure, the partial differential of the total energy per atom *E* to the volume per atom *V*_o_ can be expressed as

7 $$ \frac{\delta E}{{V_{0} }} = \frac{{c_{{{11}}} }}{2}\left( {\varepsilon_{1}^{2} + \varepsilon_{2}^{2} + \varepsilon_{3}^{2} } \right) + c_{{{12}}} \left( {\varepsilon_{2} \varepsilon_{3} + \varepsilon_{3} \varepsilon_{1} + \varepsilon_{1} \varepsilon_{2} } \right) + \frac{{c_{{{44}}} }}{2}\left( {\varepsilon_{4}^{2} + \varepsilon_{5}^{2} + \varepsilon_{6}^{2} } \right); $$

where $\varepsilon_{i}$ with *i* = 1–6 are the components of the strain tensor along the crystal axes with unit vectors along the [100], [010], and [001] directions; *c*_ij_ are the elastic stiffness tensor components^23,24,45^. In cubic symmetry, only *c*_11_、*c*_22_、*c*_44_ are independent. For tetragonal strain, there is no shear strain. Since the strain components are in the crystal axis system, therefore we have $\upvarepsilon _{1} =\upvarepsilon _{2} = \delta a/a, \;\upvarepsilon _{3} = {\delta c}/{\text{c}},\;\upvarepsilon _{4} =\upvarepsilon _{5} =\upvarepsilon _{6} = 0$ ^24^. After substituting the strain component into Eq. (7), one can obtain

8 $$ \frac{\delta E}{{V_{0} }} = \left( {c_{{{11}}} + c_{{{12}}} } \right)\varepsilon_{1}^{2} + 2c_{{{12}}} \varepsilon_{1} \varepsilon_{3} + \frac{{c_{{{11}}} }}{2}\varepsilon_{3}^{2} . $$

The strain energy density can be expressed with no cross-terms by taking the quadratic function of two particular strain components,

9 $$ \frac{\delta V}{V} = 2\frac{\delta a}{a} + \frac{\delta c}{c},\;\frac{{\delta \left( {c/a} \right)}}{{\left( {c/a} \right)}} = \frac{\delta c}{c} - \frac{\delta a}{a}; $$

with $\delta a = a - a_{0}$, $\delta c = c - c_{0}$, and $\delta V = V - V_{0}$ ^24^. Equation (8) can be expressed as

10 $$ \frac{\delta E}{{V_{0} }} = \frac{B}{2}\left( {\frac{\delta V}{V}} \right)^{2} + \frac{{{\text{2G}}}}{3}\left( {\frac{{\delta \left( {c/a} \right)}}{c/a}} \right)^{2} ; $$

where the bulk modulus $B\left( { = \frac{{c_{{{11}}} + {\text{2c}}_{{{12}}} }}{3}} \right)$ describes the compressibility of a substance and the shear modulus $G = \left( {\frac{{c_{{{11}}} - c_{{{12}}} }}{2}} \right)$ describes the relation between shear stress and shear strain. By substituting the literature values for the elastic stiffness tensor components^25,46^, the values for both the bulk modulus *B* and the shear modulus *G* can be obtained, and are tabulated in Table 1. By substituting the bulk modulus *B* and the shear modulus *G* from Table 1 and the lattice parameters *a* and *c* from Fig. 3b into Eq. (10), it becomes possible to evaluate the strain energy density versus the Fe thickness for Fe/Ir(111), as shown in Fig. 5b (black squares). For Fe/Ir(111) thinner than 3 ML, the strain energy density is nearly a constant because of the pseudomorphic growth of Fe layers. For thicker Fe layers, the structure of Fe is restored to bcc. The corresponding strain energy density decreases dramatically to a smaller value. Considering the thickness of the strained layer, the interfacial strain energy *E*_i_ can be expressed as

11 $$ E_{{\text{i}}} = \frac{\delta E}{{V_{0} }} \cdot t_{{{\text{Fe}}}} ; $$

where *t*_Fe_ is the layered distance of deposited Fe layers. From the nonzero intercept for the extrapolation of the Kerr intensity in Fig. 1a, the fcc structure for the first three ML remains the same as the Fe thickness increases to above 3 ML. It is necessary to use the interlayered distance of the fcc-Fe in the calculations. The interfacial strain energy *E*_i_ versus the Fe thickness can then be evaluated, as shown in Fig. 5c (black squares). From the literature^42,47^, the surface energy of Fe(110) facet is 2.430 J/m^2^. A critical thickness where the increasing interfacial strain energy exceeds the surface energy occurs at a thickness of 3.5 ML as derived from Eq. (11). The good agreement for critical thicknesses from the experimental measurements and calculation results suggests that the limited distortion mechanism can be useful for correlating the increasing of the interfacial strain energy *E*_i_ and the surface energy *E*_s_.

**Table 1:** Elastic stiffness tensor components^25,46^, bulk modulus *B*, and the shear modulus *G* in the unit of Mbar (10^11^ N/m^2^) for bcc-Fe and fcc-Fe.

	*c*_11_	*c*_12_	*c*_44_	*B*	*G*
bcc-Fe	2.431	1.381	1.219	1.731	0.525
fcc-Fe	2.1	1.61	1.38	1.77	0.245

In order to confirm that the limited distortion mechanism is not a special case only for Fe/Ir(111), we applied this approach to other systems. As an example of Fe grown on a Pt(111) surface, layer-by-layer growth occurs at thicknesses of up to 3 ML^14^. From an analysis of STM images, the pseudomorphic growth of fcc-Fe of the first two MLs was obtained^48^. The phenomenon of the formation of coherent regions followed by island growth for Fe/Pt(111) is similar to that of Fe/Ir(111). Our previously reported LEED data for 2 ML Fe/Pt(111) showed that the in-plane lattice parameter and interlayer distance are 2.78 Å and 2.07 Å, respectively^49^. Substituting the values for the layered distance and in-plane lattice parameter into Eqs. (3) to (6) and following the above discussions, the interfacial strain energy *E*_i_ versus the Fe thickness for Fe/Pt(111) can be evaluated as shown in Fig. 5c (blue circles). By increasing the Fe thickness, the calculated interfacial strain energy increases and eventually approaches the value for the surface energy of Fe(110) at a thickness of around 2 ML Fe/Pt(111). A smaller critical thickness of 2 ML for Fe/Pt(111) can be explained by a larger lattice misfit for Fe/Pt(111) as compared to a Fe/Ir(111) system. This result clearly shows that the limited distortion mechanism can be further applied to other system.

## Conclusions

Structures and strain accumulation for ultrathin Fe/Ir(111) were investigated. We demonstrate a strain driven phase transition in which fcc-Fe is transformed to a bcc phase. By substituting the bulk modulus and the shear modulus and the experimental results of lattice parameters in cubic geometry, we obtain the strain energy density for different Fe thicknesses. Considering the thickness of the strained layer, interfacial strain energy versus the Fe thickness can then be evaluated. A limited distortion mechanism is proposed for correlating the increasing interfacial strain energy and the surface energy. The calculation results, which indicate that the strained layers undergo a phase transition to the bulk structure above a critical thickness, are entirely consistent with the experimental measurements. In additional, a smaller critical thickness of 2 ML for Fe/Pt(111) can be explained by a larger lattice misfit for Fe/Pt(111) as compared to a Fe/Ir(111) system. . The strain driven phase transition and mechanism presented herein provide a fundamental understanding of strain accumulation at the bcc/fcc interface.
